# Crop and Semi-Natural Habitat Configuration Affects Diversity and Abundance of Native Bees (Hymenoptera: Anthophila) in a Large-Field Cotton Agroecosystem

**DOI:** 10.3390/insects12070601

**Published:** 2021-07-01

**Authors:** Isaac L. Esquivel, Katherine A. Parys, Karen W. Wright, Micky D. Eubanks, John D. Oswald, Robert N. Coulson, Michael J. Brewer

**Affiliations:** 1 Department of Entomology, Texas A&M University, College Station, TX 77843, USA; kwright@tamu.edu (K.W.W.); m-eubanks@tamu.edu (M.D.E.); j-oswald@tamu.edu (J.D.O.); r-coulson@tamu.edu (R.N.C.); mjbrewer@ag.tamu.edu (M.J.B.); 2 Department of Entomology, Texas A&M AgriLife Research, Corpus Christi, TX 78406, USA; 3 Pollinator Health in Southern Crop Ecosystems Research Unit, USDA-ARS, Stoneville, MS 38732, USA; katherine.parys@usda.gov

**Keywords:** native bees, agroecosystems, landscape structure, cotton

## Abstract

**Simple Summary:**

Commercial cotton growing systems are one of the most intensely managed, economically, and culturally important fiber cropping systems worldwide. The composition and configuration of crop species and semi-natural habitat can have significant effects on ecosystem services such as pollination. Here, we investigated the local-scale effect on the arrangement of different crop fields and surrounding semi-natural habitat in a large-field commercial cotton system on the diversity and abundance of native bee pollinators. Using bee bowl traps at crop interfaces (cotton grown next to cotton, sorghum, or semi-natural habitat along with a natural habitat comparator), we found a total of 32 bee species in 13 genera across 3 families. The most abundant native bee was *Melissodes tepaneca* Cresson (>4000 individuals, ~75% of bees collected). A higher number of individuals were found in all cotton–crop interfaces compared to the cotton next to semi-natural habitat or natural habitat alone. Native bee communities were also found to be influenced by the crop interface. Communities of native bees in the cotton–crop interfaces tended to be more consistent in the number of bees and number of bee species. While cotton grown next to semi-natural habitat had a more diverse array of bees, the number of bees collected varied. These data suggest that native bee communities persist in large-field commercial cotton growing systems. Select species dominate (i.e., *M.* *tepaneca*) and thrive in this large-field cotton system where cotton–crop interfaces are key local landscape features. These data have implications for potential pollination benefits to cotton production. The findings contribute to a discussion regarding the role of large-field commercial cotton growing systems in conserving native bees.

**Abstract:**

The cotton agroecosystem is one of the most intensely managed, economically and culturally important fiber crops worldwide, including in the United States of America (U.S.), China, India, Pakistan, and Brazil. The composition and configuration of crop species and semi-natural habitat can have significant effects on ecosystem services such as pollination. Here, we investigated the local-scale effect of crop and semi-natural habitat configuration in a large field (>200 ha in size) cotton agroecosystem on the diversity and abundance of native bees. The interfaces sampled included cotton grown next to cotton, sorghum or semi-natural habitat along with a natural habitat comparator. Collections of native bees across interface types revealed 32 species in 13 genera across 3 families. Average species richness metrics ranged between 20.5 and 30.5, with the highest (30.5) at the interface of cotton and semi-natural habitat. The most abundant species was *Melissodes tepaneca* Cresson (>4000 individuals, ~75% of bees collected) with a higher number of individuals found in all cotton–crop interfaces compared to the cotton interface with semi-natural habitat or natural habitat alone. It was also found that interface type had a significant effect on the native bee communities. Communities of native bees in the cotton–crop interfaces tended to be more consistent in species richness and abundance. While cotton grown next to semi-natural habitat had higher species richness, the number of bees collected varied. These data suggest that native bee communities persist in large-field cotton agroecosystems. Selected species dominate (i.e., *M.* *tepaneca*) and thrive in this large-field cotton system where cotton–crop interfaces are key local landscape features. These data have implications for potential pollination benefits to cotton production. The findings also contribute to a discussion regarding the role of large-field commercial cotton growing systems in conserving native bees.

## 1. Introduction

Anthropogenic intensification has resulted in a simplification of agricultural landscapes or agroecosystems, leaving small fragments of natural habitats among a few dominant crop species. This modification to the landscape is relevant to the agroecosystems of the United States of America (U.S.), Brazil, and other regions where cotton production is intensive and composed of large fields. This study considers whether, even in simplified agroecosystems, local-scale effects of the configuration of crop species and semi-natural habitat can have significant effects on ecosystem services such as natural pest control and pollination. Semi-natural habitats provide essential resources such as pollen, nectar, alternative hosts, and over-wintering sites for natural enemies and pollinators [1]. These can bolster the ecosystem services they provide, which is important for pest management and crop production [1,2]. In addition, cropland may provide pollination resources but may be limited given the simplification of annual cropping systems.

The cotton agroecosystem is one of the most intensely managed, economically, and culturally important fiber crops worldwide. In the U.S., more than 4.5 million ha of cotton were cultivated in 2017, all of which were planted in the southern U.S. Cotton Belt, spanning from Virginia to California [3]. Texas produces roughly 45% of the U.S. cotton, including where this study is located [3]. Under agricultural intensification, as seen in our model cotton agroecosystem, field sizes commonly exceed 240 hectares.

Several species of non-*Apis* bees have been observed visiting and nesting in cotton fields frequently [4], and cotton as a mass flowering crop may aid bee conservation [5]. Cotton is a perennial plant in the family Malvaceae, which is managed as an annual crop for fiber production. The plants have large flowers that produce large quantities of pollen and nectar available to many insects, including bees. The availability of mass flowering crops such as cotton across agricultural landscapes often has a positive impact on the density of generalist, native bee species, and possibly biodiversity [5].

Knowledge on abundance and diversity of native bees is also relevant to cotton production. Much of the literature on bees and their activity in U.S. cotton is roughly 30 years old. Most of this work was done in Arizona and the adjoining Texas Panhandle to identify potential bee species that could be developed and managed for economically feasible hybrid cottonseed production with special attention to the non-native and intensively managed *Apis mellifera* L. [6]. Although cotton is generally considered self-pollinating, previous studies suggest it does benefit from cross-pollination [7]. Cusser et al. [7] documented increased seed cotton weights from bolls produced from flowers that were pollinated by hand compared to bolls from flowers that were self-crossed. Further, a native bee, *Melissodes tepaneca* (Cresson), has also been shown to provide services pollination services increasing cotton yields [8]. There is a need to understand the current pollinator community within the cotton agroecosystem and its relationship to landscape structure in this large system.

Large-system and simplified agricultural production and biodiversity conservation have been traditionally viewed as incompatible [3]. Concepts of a biodiversity-ecosystem service relationship within the context of large-simplified agricultural systems can benefit from considering the influence of landscape structure and composition given an individual insect species resource needs and foraging capabilities [9]. Landscape structure may affect the diversity of pollinator communities in cotton, including native bees, beetles, and syrphid flies. Consistent with this traditional view, Cusser et al. [7] found higher pollinator diversity and abundance in cotton fields with higher amounts of semi-natural habitats were present.

Cotton producers growing cotton for fiber do not currently utilize managed pollinators (e.g., *Apis*, *Bombus*) to aid production nor use conservation practices that promote the visitation of wild pollinator communities. Further, corbiculate bees (bees with pollen baskets, primarily *Apis* and *Bombus* in the U.S.) cannot effectively collect pollen from plants in the family Malvaceae and are often frequently observed visiting cotton flowers for nectar resources [10]. This loss of effectiveness is often attributed with the length of the spines on cotton pollen which physically interfere with the pollen aggregating process used by honey bees and bumble bees [11]. If cotton is to benefit from pollinators, then native, unmanaged bees such as *Melissodes tepaneca* (Cresson) and native bee diversity are likely to play a significant role [8].

We hypothesize that local-scale crop and semi-natural habitat configuration in a large field (>200 ha in size) cotton agroecosystem affects the diversity and abundance of native bees. The objective of this study was to investigate the diversity and abundance of native bee pollinators in a model large-field cotton agroecosystem with consideration of landscape configuration of crops and semi-natural habitat at the local scale. The potential of native bee pollinators benefiting from cotton is considered using the results of this study and placed in the context of potential native bee pollination benefits to cotton production while potentially conserving native bees found in the simplified large-field cotton agroecosystem.

## 2. Materials and Methods

### 2.1. Study System

The study area was approximately 12,000 hectares of a large commercial farming operation managed by one private entity that provided continuous access and agronomic management records. This commercial farming operation was located within the south Texas coastal cotton-growing region. The main crops consisted of an annual rotation of upland cotton varieties and sorghum at an approximately 1:1 ratio and were grown following standard agronomic practices for the Texas coastal region [12]. The study area was juxtaposed on the natural gulf prairie habitat consisting of shrubland and a network of rivers, streams, and creeks that drain into the Gulf of Mexico. Semi-natural habitats were relatively sparse and primarily associated with a natural and partially augmented (i.e., drainage ditches along cultivated fields) water system flowing into the Gulf of Mexico. Agricultural intensification in our study area also included tillage for planting and weed control and insecticide applications for various cotton and sorghum pests, although insecticide use was substantially reduced from previous decades [13].

**Figure 1:**
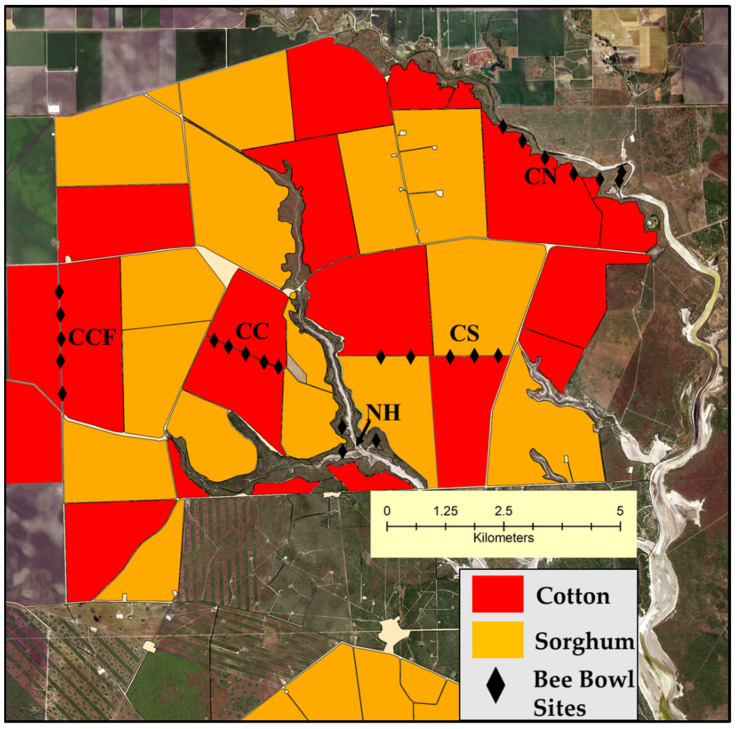
Map of study area: large-scale commercial cotton agroecosystem where average field sizes often exceeded 200 hectares. Main rotating crops consist of cotton and sorghum. Semi-natural habitats were relatively sparse and primarily associated with a natural and partially augmented (i.e., drainage ditches along cultivated fields) water system flowing into the Gulf of Mexico. Black diamonds (for visualizations purposes) represent the area where individual bee bowl sites along the 1 km to 1.5 km distances along an interface were placed (3 interface types for 2017 and 5 interface/habitat types in 2018). Bee bowls were randomly placed along the length of the interfaces during each of the four sampling events. Note: this crop layout is for 2018.

Field sizes and shapes ranged from 200 to 600 hectares and varied from high curvilinearity with large edge-to-area ratios to simple polygons with low edge-to-area ratios (Figure 1). This allowed for bee collections to be taken at five different cotton–crop or cotton–semi natural habitat interfaces, along with a comparison with a natural habitat alone. The interface was defined as the margin between two crop fields or crop fields and semi-natural habitats. Fields were separated by a narrow dirt road large enough for farm equipment to pass through. Interfaces were selected from multiple fields of a large farm operation that were available to the study. Assignment of interfaces was random within the constraint of the arrangement of fields and non-crop vegetation allowed by farm management (Figure 1). Bee collections occurred in 2017 and 2018, with sample fields changing between the years and specific sampling locations (sites) of the selected interfaces of these fields changing within years. Three interfaces were considered in 2017: A cotton field grown next to another cotton field (designated as cotton–cotton, CC in graphics), cotton grown next to sorghum (designated as cotton–sorghum, CS in graphics), and cotton grown next to semi-natural habitat (designated as cotton–natural habitat, CN in graphics). In 2018, in addition to these three interfaces, two more treatments were considered. These were cotton grown next to another cotton field more than 1 km away from semi-natural habitat (designated as cotton–cotton-far, CCF in graphics) and a natural habitat alone comparison was at least 200 m from a cotton field edge (designated as natural habitat, NH in graphics).

### 2.2. Bee Collection and Processing

Bee bowls (i.e., modified pan traps) were used for collecting native bees. Bee bowls are the most cost-effective and simplest method to monitor bees in agricultural systems [14,15]. These consisted of three ~100 mL (3.25 oz) Solo cups, painted either flat white, fluorescent blue, or fluorescent yellow. We note that bee bowls are known to be biased toward smaller bees as opposed to larger bees. However, bees collected in bee bowls were often the ones seen visiting cotton during the collection period (I.L.E. Pers. Obs.). The three bee bowls were individually fastened to shelving brackets held with industrial strength Velcro and attached to T-posts staked into the ground. The bee bowls were positioned at canopy level within the crop-free area of the interface closer to cotton as not to interfere with farm equipment. Specific sites were randomly selected along the long crop–crop interfaces or within the natural habitat alone. In 2017, three sites per three selected crop interfaces (Cotton–Cotton, Cotton–Sorghum, Cotton–Semi Natural) were sampled, totaling 27 individual bee bowls (9 sites) for each sampling event. In 2018, five sites at all interfaces (Cotton–Cotton, Cotton–Sorghum, Cotton–Semi Natural, Cotton–Cotton-Far, Natural–Habitat) were sampled, totaling 75 individual bee bowls (25 sites) per sampling event. Traps were set out at each site at first bloom and sampled weekly for a period of four weeks for each year. Each week, bee bowl sites were randomly placed along the specified interfaces. The specific length of the interface for sampling varied from 1 km to 1.5 km depending upon field access. Sampling began in the morning, and bee bowls were collected 24 h later or soon thereafter as weather and road conditions allowed. Because of the rapid growth of cotton and the random repositioning of bee bowls for each sampling event along the long interface of designated types, sampling events were considered independent and used as the unit of replication.

Once bees were collected, specimens were temporarily stored in 70% ethanol, then pinned and labeled following curatorial best practices. Bees were processed by sorting specimens into morphotypes and later identified to genus using general keys [16,17]. Following is a list of genera and corresponding primary literature used for identifications to species: *Agapostemon* [18], *Anthophora* [19], *Augochlora* [20,21], *Augochlorella, Ceratina* [22,23], *Diadasia* [24], *Halictus* [25], *Lasioglossum* [26,27], *Megachile* [28], *Melissodes* and *Svastra* [29,30,31], *Nomia* [32], and *Xylocopa* [33]. Specimens in the genera *Ceratina* and *Lasioglossum* (*Dialictus*) were not identified to species and left with morphospecies designations due to taxonomic uncertainty within the region.

### 2.3. Analyses

In order to evaluate the adequacy of our bee bowls in capturing the native bee fauna in the cotton agroecosystem, species accumulation curves were produced using the function ‘specaccum’ in the R package BiodiversityR using the expected ‘Coleman’ richness [34,35]. To evaluate species richness, we used a ‘chao1’ estimator to evaluate species richness within sites across the various field interfaces in the R package ‘Vegan’ [35]. Species richness values were normally distributed and were analyzed further using a standard one-way analysis of variance model. A mixed-model analysis of variance was used to detect differences in bee abundance across interface/habitat types. Interface/habitat was set as a fixed effect. Each year was analyzed separately due to the difference in interfaces considered each year. The multiple sites randomly placed along the length of an interface were added as a random effect to partition the variability within sites of an interface from the variances between interfaces using the function ‘lmer’ in the R package lme4 [36]. The variance partitioning was conducted because of the randomization of individual sites between sampling events was restricted to the 1 km to 1.5 km distances of interfaces, that may have resulted in less variation between sampling events (i.e., a restriction of randomization occurred due to our interest in specific interfaces in a large-field production setting using the same agronomic procedures). The bee taxa considered were the most common native bees across the three (2017) and five (2018) interface types, as well as total bee abundance across all species/morphospecies. After the variance for site was partitioned out and if a significant effect of the interface was found, we further compared abundances across interfaces and the natural habitat comparison with Tukey’s HSD means comparisons at the α = 0.05 significance level.

To characterize the overall diversity of the native bee community within the cotton agroecosystem, data from both 2017 and 2018 were used. As noted above, analyses were conducted separately for each year. Community analyses were conducted using R version 3.6.3 “Holding the Windsock” using packages Vegan and ggplot2 [37,38,39,40]. Data ordination was performed using non-metric multidimensional scaling (NMDS) of Bray–Curtis dissimilarities using Vegan and graphed in ggplot2 to visualize sample and species relationships in a low-dimensional space. Briefly, NMDS arranges points to maximize the rank-order correlation between real-world distances (Bray–Curtis) of species data between sites. It plots the pair-wise dissimilarity between objects in ordination space. Objects (in this case, sites) that are ordinated closer to one another are more similar in species composition and abundance than those further apart. An analysis of similarity test (ANOSIM) using the Bray–Curtis similarity matrix obtained from 999 permutations was used to test differences between the native bee community at different interfaces using the function ‘anosim’ in the R package Vegan [38,39]. The ANOSIM analysis produces a test statistic ‘R’ that compares the mean of ranked dissimilarities between groups to the mean of ranked dissimilarities within groups. A *p*-value of the R statistic is determined by multiple permutations of the group membership to obtain the null distribution of the R statistic [40]. Comparing the position of the observed R-value to the null distribution allows an assessment of the statistical significance of R [40]. R values range from −1 to 1. Those closer to 1 indicate strong dissimilarity between groups, whereas those closer to −1, indicate similarity within the groups, in our case sites in different interfaces.

## 3. Results

**Figure 2:**
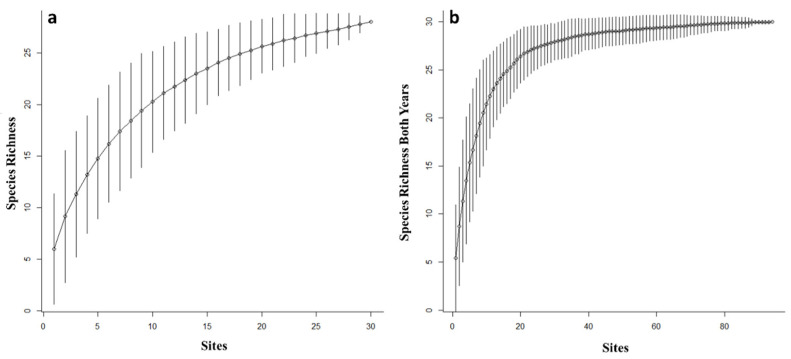
Species accumulation curves from bee bowl collections conducted in 2017 (**a**) and combined 2017 and 2018 (**b**) across crop and semi-natural habitat interfaces in a large-scale cotton agroecosystem.

**Figure 3:**
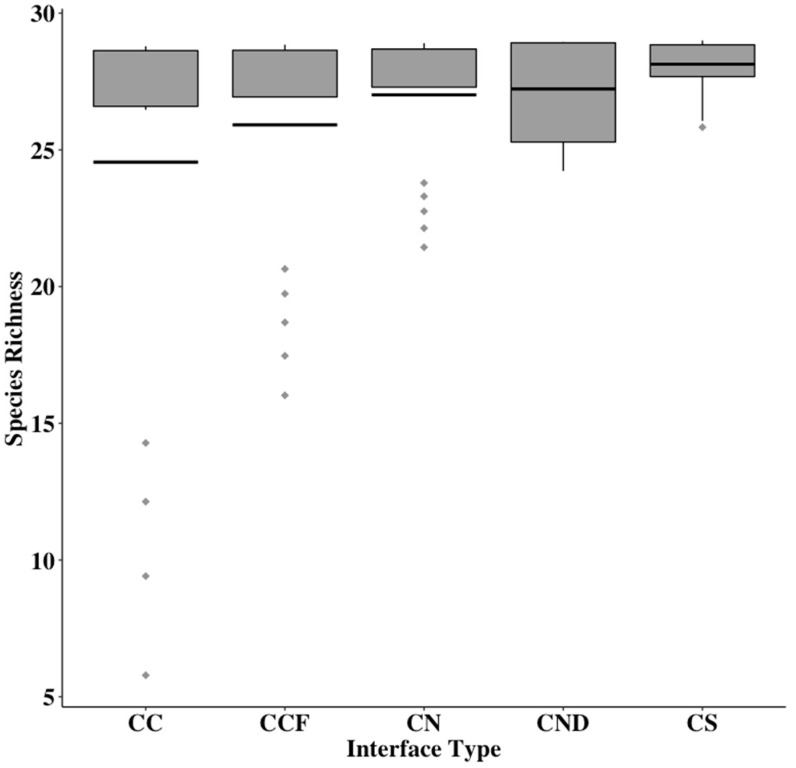
Species richness calculated from bee-bowl collections in both years (2017 and 2018 combined) shown as boxplots across interfaces from bee-bowl collections in a large-scale cotton agroecosystem. Interface types were sites between a cotton field and another cotton field (CC), a sorghum field (CS), semi-natural habitat (CN), another cotton field 1 km away from semi-natural habitat (CCF) and semi-natural habitat alone (NH). The solid black bar gives treatment mean, boxes give the 1st and 3rd quartiles, and diamonds show outliers.

In 2017, a total of 897 native bees (excluding 21 *A. mellifera*) were collected using bee bowls, representing a total of 28 species. Inspection of species accumulation curves for the 2017 native bee community increased at a high rate and began to level-off as more sites and sampling events were added, although a plateau was not reached (Figure 1). This led to an increased sampling effort in 2018, including the sampling of two additional interfaces. In 2018, a total of 4666 native bees (excluding 26 *A. mellifera*) were collected, representing 32 species. *Apis mellifera* were excluded from analyses as we were interested in native bees. Further, managed honey bees are not purposefully used in cotton management in this model system. The combined 2017 and 2018 species accumulation curve increased exponentially, reaching a plateau indicating that the sampling effort was effective in capturing the native bee species in the area using the bee bowls (Figure 2b). Across both years, a total of 5563 specimens were collected, representing 32 species in 13 genera and three families (Table 1). Average species richness across sites per interface type ranged from 20.5 to 30.5 combined across both years (Figure 3). The two most abundant taxa were *Melissodes* (4233 individuals) consisting of two species, and the subgenus *Lasioglossum* (*Dialictus*) (989 individuals) composed of 12 species/morphospecies across both years (Table 1). The number of native bees collected at different cotton–crop and cotton–semi-natural interfaces in a large-scale cotton agroecosystem.

**Figure 4:**
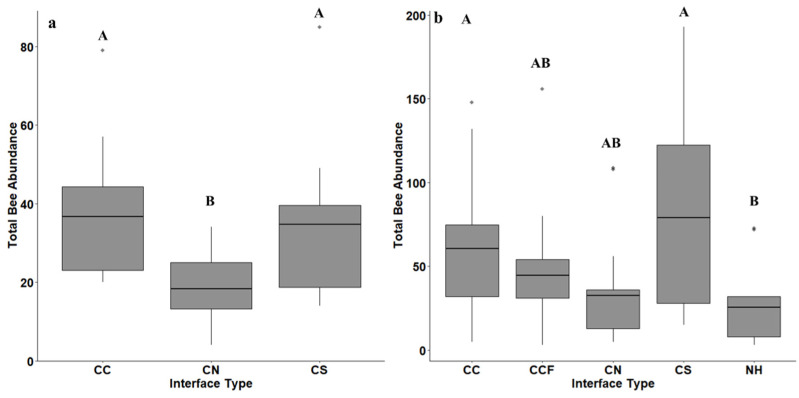
The average abundance of all native bees across across crop and semi-natural interfaces taken from bee-bowl collection conducted in 2017 (**a**) and 2018 (**b**) shown as boxplots. The interface types were sites between a cotton field and another cotton field (CC), a sorghum field (CS), semi-natural habitat (CN), another cotton field 1 km away from semi-natural habitat (CCF) and semi-natural habitat alone (NH). The solid black bar gives treatment mean, boxes give the 1st and 3rd quartiles, and diamonds show outliers. Abundances across interface types were compared using Tukey’s HSD means procedure with letters differing across means indicating significant differences.

When investigating the effect of interfaces on the total amount of native bees collected in both 2017 (F = 4.26; df = 2, 27; *p* = 0.02) and 2018 (F = 3.75; df = 4, 69; *p* = 0.008), more native bees were collected at crop–crop interfaces. In 2017, more native bees were collected on average at the interface of cotton–sorghum and cotton–cotton compared to the interface of a cotton field-semi-natural habitat (Figure 4a). In 2018, more native bees on average were collected at the interface of a cotton–sorghum, and cotton–cotton, compared to cotton–natural habitat alone (Figure 4b). The interfaces of a cotton–cotton-far and natural habitat were intermediate in abundances (Figure 4b).

**Figure 5:**
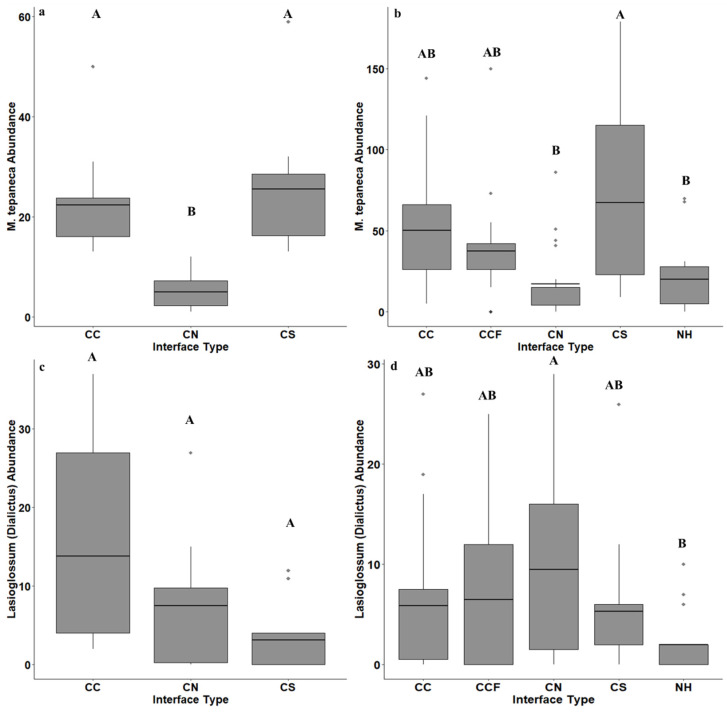
The average abundance of *Melissodes tepaneca* (**a**,**b**) and *Lassioglossum* (*Dialictus*) spp. (**c**,**d**) across crop and semi-natural interfaces taken from bee-bowl collection conducted in 2017 and 2018 in a large-scale cotton agroecosystem. The interface types were sites between a cotton field and another cotton field (CC), a sorghum field (CS), semi-natural habitat (CN), another cotton field 1 km away from semi-natural habitat (CCF) and semi-natural habitat alone (NH). The solid black bar gives treatment mean, boxes give the 1st and 3rd quartiles, and diamonds show outliers. Abundances across interface types were compared using Tukey’s HSD means procedure with letters differing across means indicating significant differences.

*Melissodes tepaneca* and *Lasioglossum* spp. were often seen visiting cotton flowers in the field frequently and *M. tepaneca* has been shown to benefit cotton yield [7,15], therefore we investigated these two species/species-groups further. *M. tepaneca* was the most abundant species in both years, with 528 individuals collected in 2017 and 3686 individuals in 2018. Significant differences in the abundance of *M. tepaneca* were detected across three interfaces in 2017 (F = 3.85; df = 2, 27; *p* = 0.03) and five interface types in 2018 (including semi-natural habitat) (F = 3.22; df = 4, 69; *p* = 0.017) (Figure 5a,b). In 2017, the interface of cotton–semi-natural habitat had the lowest abundance of *M. tepaneca* on average compared to the interfaces of a cotton–cotton, and cotton–sorghum (Figure 5a). In 2018, more *M. tepaneca* were collected on average at the interface of a cotton–sorghum compared to the interface of cotton–semi-natural habitat or natural habitat alone. Abundance of *M. tepaneca* in the remaining two interfaces were intermediate in abundance, including the cotton–cotton-far interface that was 1 km from natural habitat (Figure 5b).

Interface type had a significant effect on the abundance of 12 *Lasioglossum* (*Dialictus*) species in 2018 (F = 4.66; df = 4, 69; *p* = 0.002) but not 2017 (F = 3.12; df = 2, 27; *p* = 0.06) (Figure 5c,d). In 2018, more *Lasioglossum* (*Dialictus*) spp. were caught on average at the cotton and semi-natural habitat interface compared to the natural habitat alone. The other interfaces had an intermediate number of *Dialictus* spp. collected (Figure 5d).

**Figure 6:**
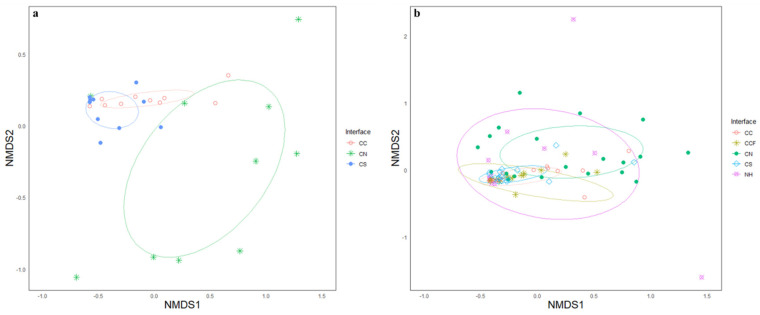
A Non-metric multidimensional scaling (NMDS) of Bray–Curtis similarity data of native bee communities in 2017 (**a**) and 2018 (**b**) across crop and semi-natural interfaces in a large-scale cotton agroecosystem. Each point is representative of a site placed according to their similarities with other sites, and ellipses representing the variance of the number of species within those sites. Widths of ellipses indicating the bee community similarity within sites at a given interface are greater than the similarities between the sites at different interfaces.

In 2017, three groups of native bee communities of the three interface types appeared distinctive, showing well-formed groups on the NMDS plot (Figure 6a). The reasonably low-stress level (0.0768) indicated a fair representation of multidimensional space. The ANOSIM analysis indicated moderate differences (*R* = 0.2914) and was overall significant (*p* = 0.001), indicating that the communities between the sampling sites across the cotton–cotton, cotton–sorghum, and cotton–semi-natural habitat interfaces were more different from one another, while the sampling sites within each interface type were more similar to each other. The width of the ellipses suggests that sites in the cotton–natural habitat interface had a higher variance in the similarity between sites (Figure 6a). In contrast, the other two interfaces were more consistent in the species found across the sites. In 2018, the NMDS ordination indicated groupings of communities between the five interface types with a reasonably low-stress score (0.0944). However, the confidence ellipses with an ANOSIM test statistic (*R* = 0.1511) showed some overlap between the interface groupings with at least some differences in the groups detected (*p* = 0.001) (Figure 6b). This is reflected in the position and the size of the ellipse groupings in the NMDS plot. For example, the natural habitat ellipse appears to overlap all the other communities of the cotton–crop and cotton–natural interfaces. This suggests that although most species are found in the natural habitat alone, the number of species caught between sampling sites across the samples were more variable. Whereas the ellipses at the interface of cotton–sorghum and cotton–cotton were rather tight, suggesting that there was consistency in the bee species collected between the sampling sites at each sampling event.

## 4. Discussion

In this large-scale cotton agroecosystem, a total of 33 species (including *A. mellifera*) were collected, with a total of 5563 native bees collected across 2017 and 2018. The majority of these bees were composed of a single species, *Melissodes tepaneca*, representing 75% of the individuals collected. These data are consistent with both past and current literature in which species in the genus *Melissodes* are found within cotton fields in various parts of U.S cotton-growing regions. For example, in experimental observations of bees visiting Georgia cotton flowers by Allard [41], roughly 80% of over 2000 visual identifications were of a single species, *Melissodes bimaculatus* (Lepeltier). *Melissodes thelypodii* (Cockerell) was also abundant in cotton of the Texas High Plains [6]. In the same study, one species in the genus *Agapostemon* (*A*. *angelicus* (Cockerell)), was found in high abundance [6]. In our study, we found three species of *Agapostemon* at detectable rates but low abundance. More recently in Texas, using hand collection methods, 37 species of bees were found in small-scale cotton systems with most field sizes below 200 ha. At this small scale, 21% out of 800 total specimens collected were *M*. *tepaneca* [7]. The genus *Melissodes* also appears to be important in cotton outside of the U.S. In Brazil, *M. nigroaenea* (Smith) has been identified as an important pollinator in small-scale cotton agroecosystems systems [42,43].

Unlike some of the studies previously mentioned, where native bee abundances and diversity were measured at smaller scale cotton fields (<50 hectares) with more diverse landscapes, our study took place within a large-scale commercial cotton agroecosystem where average field sizes often exceeded 200 hectares. Despite the dominance of the crop–crop interfaces and other aspects of agricultural intensification, the bee communities were similar in terms of species richness at different interfaces of this highly structured large-scale cotton agroecosystem, suggesting these crops such as cotton can play a role in future conservation efforts. Compared to other studies [7,42,43], where increasing native bee diversity and richness in more diverse landscapes were seen, our findings are somewhat unexpected and lent support to the hypothesis that local-scale crop and semi-natural habitat configuration in a large field (>200 ha in size) cotton agroecosystem affects the diversity and abundance of native bees within the system.

A recent review article investigating potential benefits of native bee pollinators in mass flowering crops such as cotton and soybean indicates a lack of baseline data of bee fauna in these systems, making it difficult to determine long term effects of agricultural intensification on bee communities [44]. A few of the abundant generalist species found here, including *Melissodes*, *Agapostemon*, *Halictus*, *Lasioglossum* (*Dialictus*), and *Nomia*, are also common in other cropping systems across North America, including corn, soybeans, hemp, and alfalfa [14,15,43,45]. This suggests that selected generalist bee species are quite resilient and adaptable to changes caused by agricultural intensification. In comparison, rare or threatened species appear to be seldom seen in agricultural fields. Common crops in large scale agroecosystems are often self or wind pollinated and therefore scarce in pollinator resources, but mass flowering crops such as cotton are an important exception. Loss of specialist bees in these systems is predicted (and consistent with our observations in cotton), but several generalist bees persist and one, *M*. *tepaneca* appears to thrive. There is clearly a need for studies to investigate all aspects of bee fauna in these systems more than the abundant generalists currently seen.

Although species composition was similar across interfaces and semi-natural habitat, more native bees overall were collected within cotton–crop interfaces. This was largely dominated by a single species (*M. tepaneca*) and species group (*Dialictus*). Specifically, *M. tepaneca* appears to have an affinity for crops as it was collected in higher amounts at cotton–crop interfaces compared to cotton–semi-natural habitat interfaces or semi-natural habitat alone. They were most abundant at cotton–sorghum interfaces in both years. Furthermore, samples taken from natural habitat alone (NH) appeared to have a lower abundance but more species were present within natural habitat. This supports the suggestion that mass flowering crops such as cotton, and possibly other resources provided by sorghum, benefited the hardy generalist species such as *M. tepaneca* in this agroecosystem. However, not all species may be able to utilize these resources and may be threatened with further loss of semi-natural habitat. This is seen in Hall et al. [46], where a few hardy generalist bee species dominated open agricultural sites compared to woody vegetative sites where bee species that prefer wooded habitat were more often found.

The average foraging distance from the nesting site to the food source for most solitary bees is between 150–600 m depending on body size [47]. In a cotton operation where fields are on average 500–1000 m wide, this suggests that at least for *M. tepaneca,* nesting occurs within the area of agricultural intensification with few remnants of semi-natural habitat. It has been documented that *M*. *tepaneca* does nest within cotton rows in Arizona cotton fields [48], which may be representative of *M*. *tepaneca* in Texas cotton fields. Several species within the genus *Melissodes* have been observed to nest in aggregations of up to 200 individual nests in semi-sandy soils, both within patches devoid of vegetation and partially covered ground with leaflitter or grass [49,50]. There is little to no literature on nesting preferences for other species found in this system, specifically those that prefer and utilize natural habitat over crop resources.

On the other hand, *Lasioglossum* (*Dialictus*) spp. were more abundant at the cotton–semi-natural habitat interface, only in 2018. Further, less *Lasioglossum* (*Dialictus*) were found in the natural habitat alone compared to the interface of cotton–semi-natural habitat. This suggests that some of the species in this group have an affinity to cotton, possibly utilizing a resource in cotton when in cultivation. This group is semi-social and highly diverse in abundance and life history. Species range from specialists to generalists, and display a variety of life histories, inclusive of parasitism, and may be uncommon to abundant [27]. More detailed research on the foraging and nesting behavior of these species in agroecosystems is warranted to explore the mechanisms that may be driving our observation that native bee community within a large-scale cotton agroecosystem persists, and some species may thrive even when crop–crop interfaces dominate semi-natural habitat.

Bee conservation efforts tend to focus on native or slightly modified habitats. In the U.S. and the European Union, there are various stewardship programs that support portions of agricultural production temporarily or permanently taken out of production to augment wildlife, preserve diversity, and aid pollinators and other organisms that provide ecosystem services and eco-tourism [51]. As stated by Tscharntke et al. [2], there has been a recent debate as to whether this type of conservation to augment ecosystem services is of limited value when considering the often-neglected influence of landscape context on local processes [52,53,54,55]. In this case, processes such as crop configuration of the cotton agroecosystem affect native bee pollinators and may off-set negative aspects of agricultural intensification. The result is selective pollinators such as *M*. *tepaneca* may persist and thrive. Further, intensified land use in agriculture and forestry is a contributor to global climate change and biodiversity loss. However, Tscharntke et al. [2] state biodiversity conservation focusing on 5% of remaining pristine natural habitats will have little value without a recognition of the contribution of the of all land-use types, including intensely managed agricultural land. In the model system studied here, stewardship of native bees appears to be present in a large cotton agroecosystem at the local field-to-field level of scale that may have implications of biodiversity conservation at a larger scale when more information on bees in seminatural habitat is known.

In a large meta-analysis of pollination services provided by native bee species, Kleijn et al. [54] found that crop-visiting native bee communities are dominated by a small number of common species that persist under agricultural expansion. They also state that many of these pollinators have the potential to be enhanced by simple conservation measures such as modifying tillage mechanisms to no-till systems. Kleijn et al. [54] state these should be the focus of conservation efforts to bolster pollination services provided to agricultural production. However, without catering for bee species that inhabit different ecological niches may lead to substantial loss in the species that rely on non-agricultural resources, affecting ecosystem function as a whole [46]. In our study, *M. tepaneca* is clearly the most abundant species in the large-field cotton agroecosystem studied. This species may benefit from simple conservation measures as well. However, given its abundance in all the cotton–crop interfaces, including cotton–cotton and cotton–cotton-far, it appears to thrive in the extant large-field system. The scale and quality of crop set-asides and conservation easements are current considerations in conservation programs [54]. Although we add one data point to the debate, a consideration in the configuration of crops and semi-natural habitat in agroecosystems and key species contributors to ecosystem services should a consideration on pursuing bee conservation, which is consistent with Kleijn et al. [54].

Specific to the cotton agroecosystem, *M*. *tepaneca* has a positive effect on cotton production by increasing lint and seed weight when flowers are exposed to *M*. *tepaneca* [8]. The findings here suggest that cotton may benefit *M*. *tepaneca* and other native bees both near and relatively far from semi-natural habitat. This complementary benefit to cotton supports consideration of a joint bee conservation and cotton productivity approach in at least this select large-scale cotton agroecosystem that has substantially modified the original natural gulf prairie habitat, and that was designed from a cotton production perspective. We pose that this system may represent a win-win for bee conservation and cotton productivity, and such a system may be a valuable learning arena to explore underlying mechanisms that benefit bee conservation and augment ecosystem service in the form of pollination [8]. Additional studies into native bee natural history in this system, such as nesting habitats and preferences, and foraging preferences of *M. tepaneca*, and other common and abundant bee species will aid in understanding of how these bees persist and possibly thrive in intensified agricultural systems of large-scale cotton production. Additional studies on the complete bee fauna in these systems will help delineate the contribution to bee health (diversity and abundance) and cotton production, which can foster more healthy agroecosystems across the landscape.

## Figures and Tables

**Table 1:** The Number of native bees species collected at different crop-crop and crop-semi-natural habitat interfaces in a large-scale cotton agroecosystem.

Year of Collection	2017			2018				
**Bees Collected by Family ^2^**	**CC ^1^**	**CS**	**CN**	**CC**	**CS**	**CN**	**CCF**	**NH**
**HALICTIDAE: Augochlorini**								
*Augochlora aurifera* (Cockerell)	1	1	0	0	0	0	0	0
*Augochlorella aurata* (Smith)	0	4	0	0	0	0	0	3
**HALICTIDAE: Halictini**								
*Agapostemon melliventris* (Cresson)	4	1	8	2	11	5	1	4
*Agapostemon splendens* (Lepeletier)	1	4	0	0	3	6	2	1
*Agapostemon texanus* (Cresson)	4	0	8	7	1	11	6	2
*Halictus (Odontalictus) ligatus* Say	2	16	1	8	7	10	4	0
*Lasioglossum (Dialictus) coactum* (Cresson)	0	7	0	5	2	3	4	0
*Lasioglossum (Dialictus) connexum* (Cresson)	2	0	0	5	3	5	8	0
*Lasioglossum (Dialictus) disparile* (Cresson)	11	8	2	6	0	7	3	0
*Lasioglossum (Dialictus)* sp. A	30	15	22	25	17	18	10	4
*Lasioglossum (Dialictus)* sp. B	68	33	21	9	15	30	19	0
*Lasioglossum (Dialictus)* sp. C	2	3	0	0	12	0	6	0
*Lasioglossum (Dialictus)* sp. D	8	0	2	8	2	1	4	8
*Lasioglossum (Dialictus)* sp. G	1	1	0	23	35	71	21	4
*Lasioglossum (Dialictus)* sp. H	2	3	1	40	34	42	14	6
*Lasioglossum (Dialictus)* sp. I	0	4	0	25	30	39	22	3
*Lasioglossum (Dialictus)* sp. J	0	1	0	29	17	31	25	1
*Lasioglossum (Dialictus)* sp. K	0	0	1	0	0	0	0	0
**HALICTIDAE: Nomiini**								
*Nomia (Acunomia) nortoni* Cresson	0	0	0	0	6	0	0	0
**MEGACHILIDAE: Megachilini**								
*Megachile (Litomegachile) brevis* Say	0	0	7	0	7	8	0	4
*Megachile (Litomegachile) lippiae* Say	1	2	0	1	0	4	0	4
*Megachile (Litomegachile) gentilis* Cresson	2	9	6	1	6	6	0	5
*Megachile (Litomegachile) policaris* Cresson	0	10	0	0	4	4	0	3
**APIDAE: Anthophorini**								
*Anthophora californica* Cresson	0	1	0	0	2	0	0	0
**APIDAE: Ceratini**								
*Ceratina (Zadontomerus)* spp.	0	0	0	1	0	9	2	9
APIDAE: Emphorini								
*Diadasia rinconis* Cockerell	0	3	0	0	0	5	0	6
*Melitoma* spp.	0	0	0	0	0	0	0	3
**APIDAE: Eucerini**								
*Florilegus condignus* (Cresson)	1	0	3	2	6	0	0	0
*Melissodes (Melissodes) communis* Cresson	2	0	6	3	6	1	1	0
*Melissodes (Melissodes) tepaneca* Cresson	223	50	255	1004	1277	360	786	259
*Svastra (Epimelissodes) obliqua* (Say)	0	8	3	0	9	7	0	0
*Svastra (Epimelissodes) petulca* (Cresson)	2	1	1	4	3	0	1	2
**APIDAE: Xylocopini**								
*Xylocopa (Notoxylocopa) tabaniformis* Smith	0	0	0	2	0	0	0	0
**Total Specimens Collected**	367	185	346	1210	1505	681	938	331

^1^ Interface types represented are a cotton field next to another cotton field (CC), a cotton field and another cotton field 1 km away from natural habitat (CCF), a cotton field next to a sorghum field (CS), a cotton field next to semi-natural habitat (CN), and natural habitat alone (NH). ^2^ Bees collected by family taken from bee bowl collections across 2017 and 2018 from A total of 5563 specimens were collected, representing 32 species in 13 genera and 3 families.
